# Supplementary figures and images for: Topological data mapping of online hate speech, misinformation, and general mental health: A large language model based study
Source: PLOS Digit Health. 2025 Jul 29;4(7):e0000935. doi: 10.1371/journal.pdig.0000935 (PMC12306733; doi:10.1371/journal.pdig.0000935)

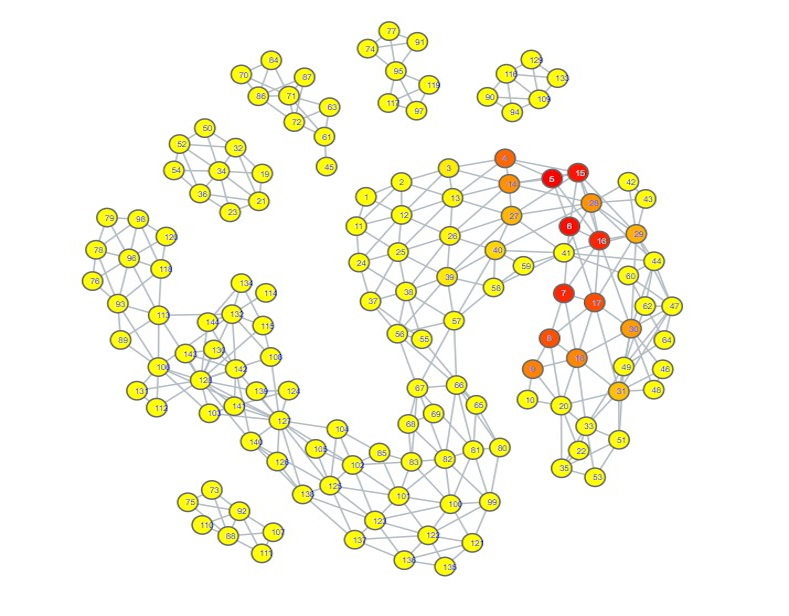

Supplement: S1 Fig — Yellow = No Attention Deficit Hyperactivity Disorder Embeddings, Red = Entirely Comprised of Attention Deficit Hyperactivity Disorder Embeddings. (TIFF) [file pdig.0000935.s001.tiff]

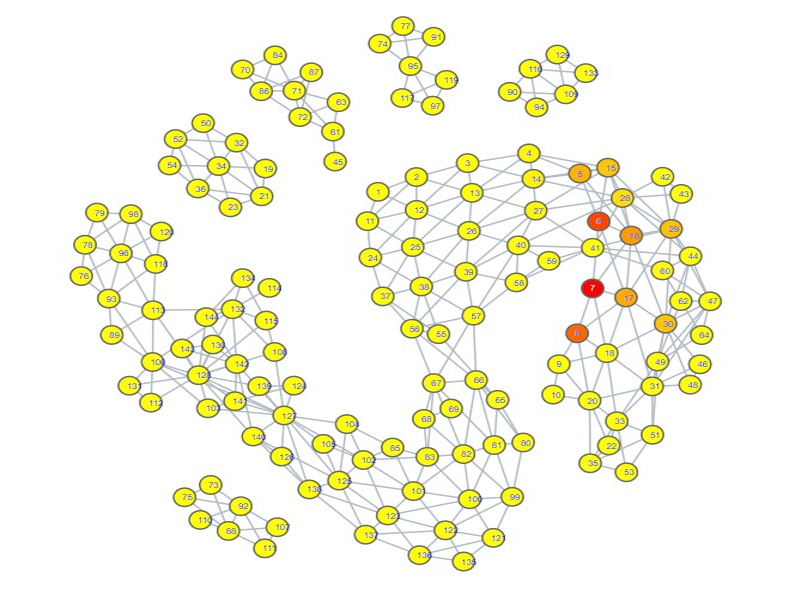

Supplement: S2 Fig — Yellow = No Anxiety Disorder Embeddings, Red = Entirely Comprised of Anxiety Disorder Embeddings. (TIFF) [file pdig.0000935.s002.tiff]

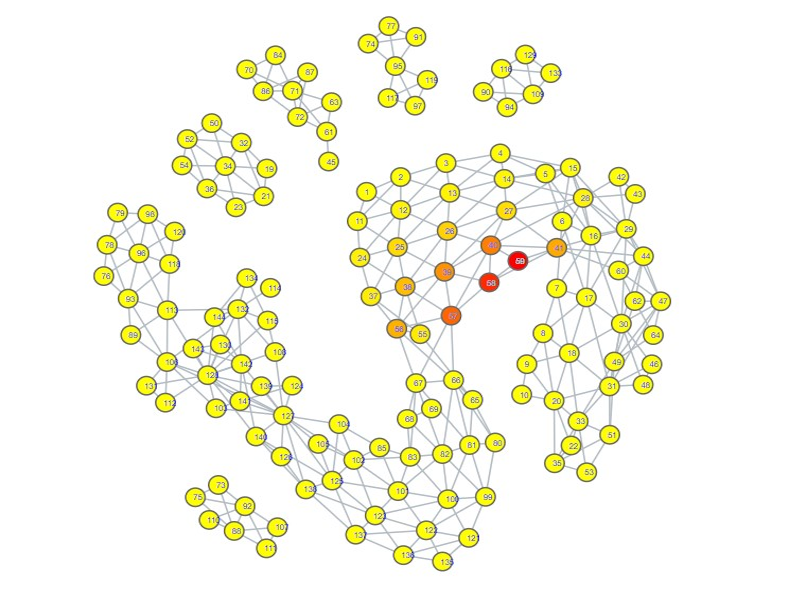

Supplement: S3 Fig — Yellow = No Autism Spectrum Disorder Embeddings, Red = Entirely Comprised of Autism Spectrum Disorder Embeddings. (TIFF) [file pdig.0000935.s003.tiff]

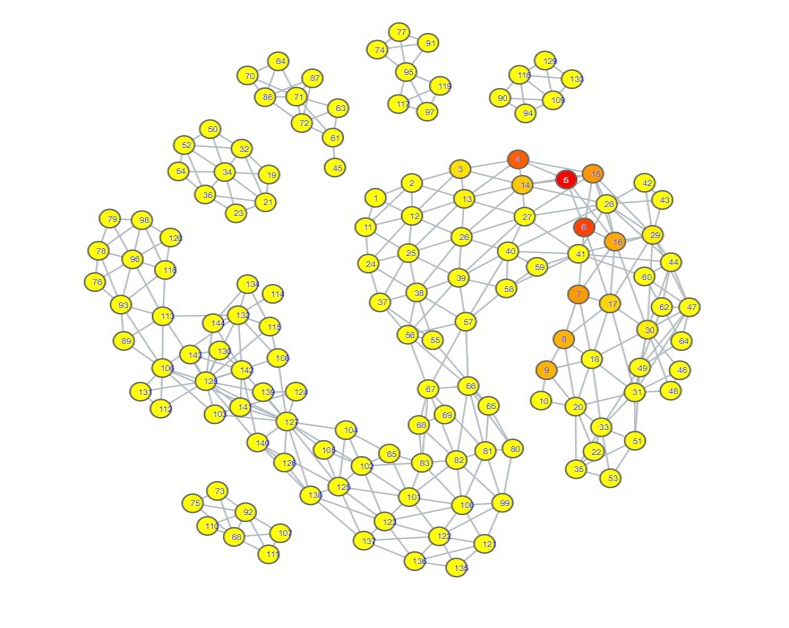

Supplement: S4 Fig — Yellow = No Bipolar Disorder Embeddings, Red = Entirely Comprised of Bipolar Disorder Embeddings. (TIFF) [file pdig.0000935.s004.tiff]

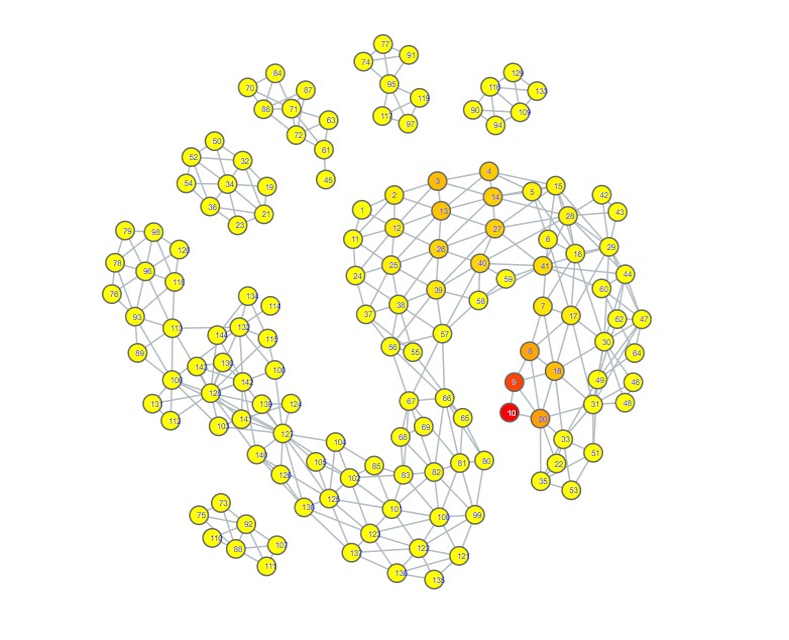

Supplement: S5 Fig — Yellow = No Depression Embeddings, Red = Entirely Comprised of Depression Embeddings. (TIFF) [file pdig.0000935.s005.tiff]

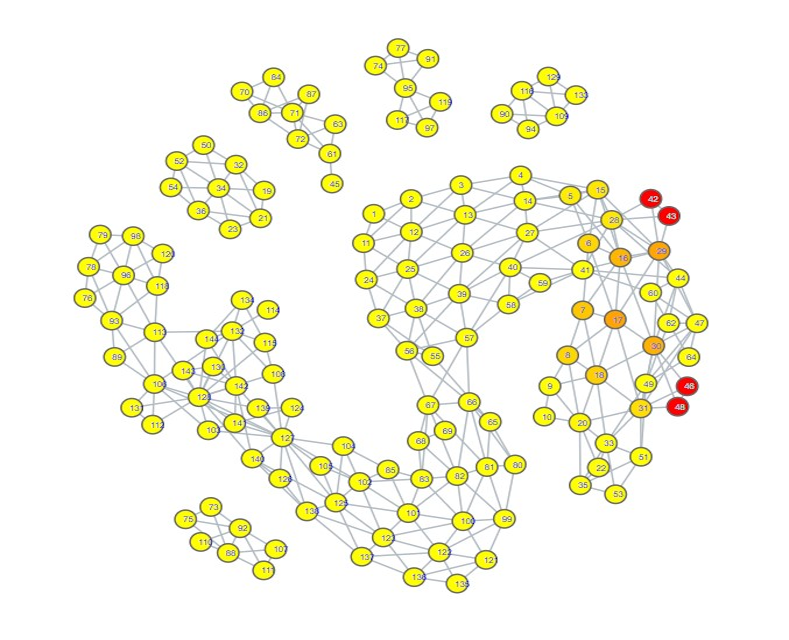

Supplement: S6 Fig — Yellow = No Eating Disorder Embeddings, Red = Entirely Comprised of Eating Disorder Embeddings. (TIFF) [file pdig.0000935.s006.tiff]

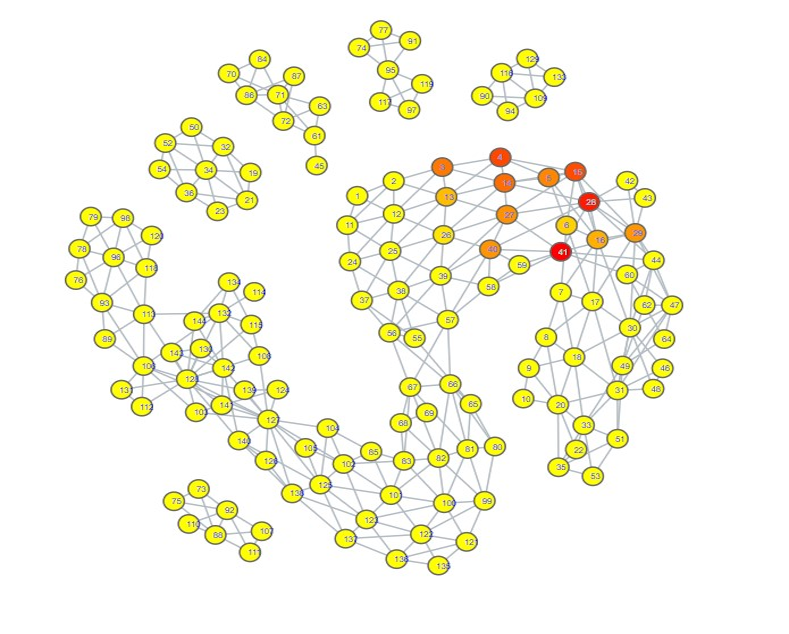

Supplement: S7 Fig — Yellow = No Obsessive Compulsive Disorder Embeddings, Red = Entirely Comprised of Obsessive Compulsive Disorder Embeddings. (TIFF) [file pdig.0000935.s007.tiff]

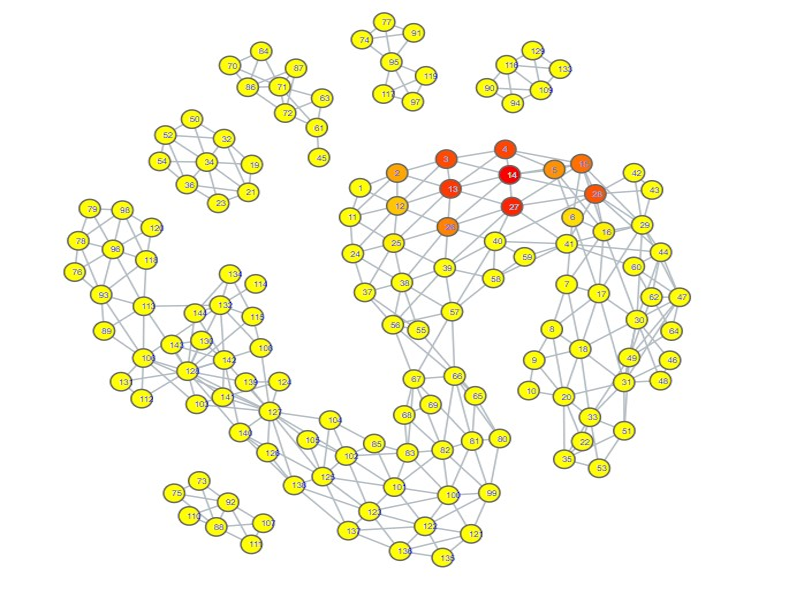

Supplement: S8 Fig — Yellow = No Post-Traumatic Stress Disorder Embeddings, Red = Entirely Comprised of Post-Traumatic Stress Disorder Embeddings. (TIFF) [file pdig.0000935.s008.tiff]

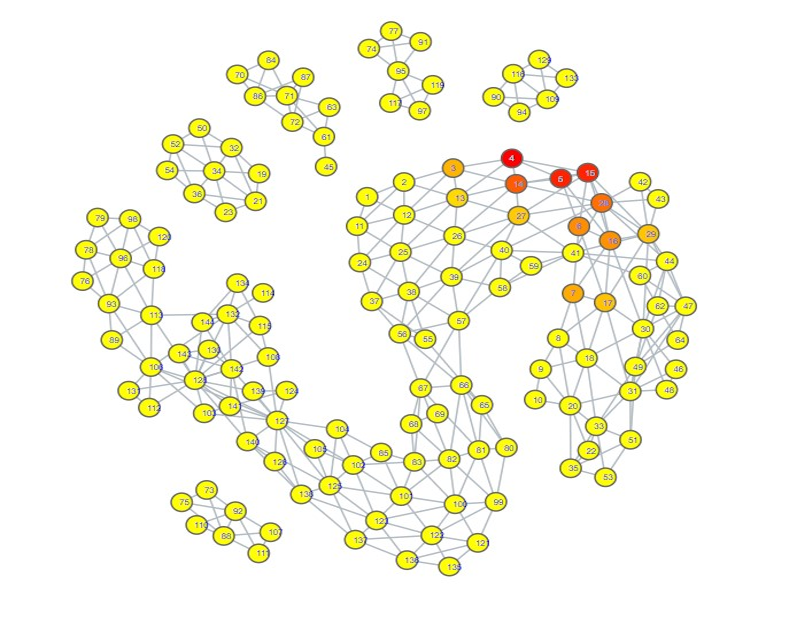

Supplement: S9 Fig — Yellow = No Schizoaffective Disorder Embeddings, Red = Entirely Comprised of Schizoaffective Disorder Embeddings. (TIFF) [file pdig.0000935.s009.tiff]

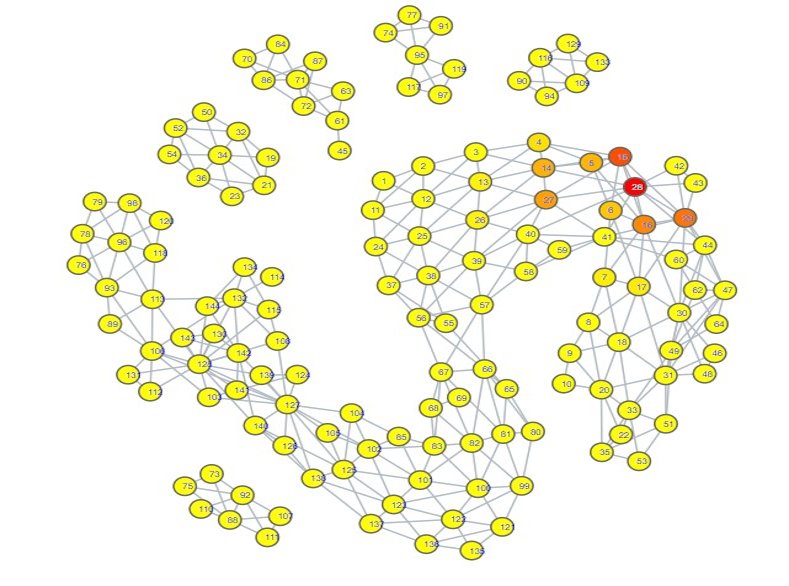

Supplement: S10 Fig — Yellow = No Schizophrenia Embeddings, Red = Entirely Comprised of Schizophrenia Embeddings. (TIFF) [file pdig.0000935.s010.tiff]

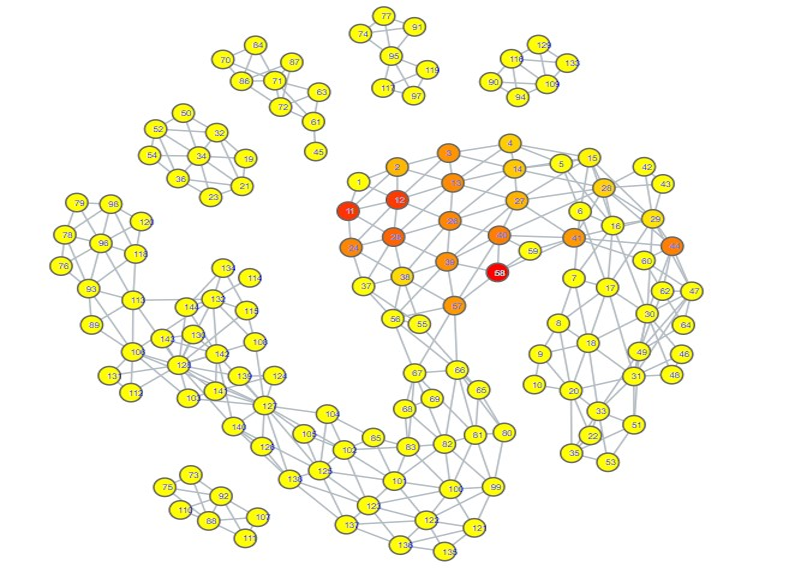

Supplement: S11 Fig — Yellow = No Schizotypal Personality Disorder Embeddings, Red = Entirely Comprised of Schizotypal Personality Disorder Embeddings. (TIFF) [file pdig.0000935.s011.tiff]

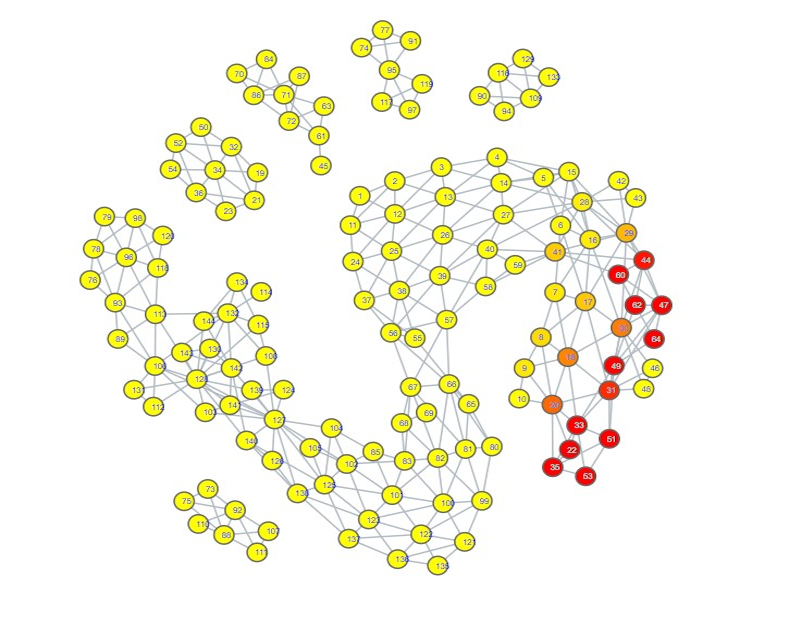

Supplement: S12 Fig — Yellow = No Substance Use Disorder Embeddings, Red = Entirely Comprised of Substance Use Disorder Embeddings. (TIFF) [file pdig.0000935.s012.tiff]

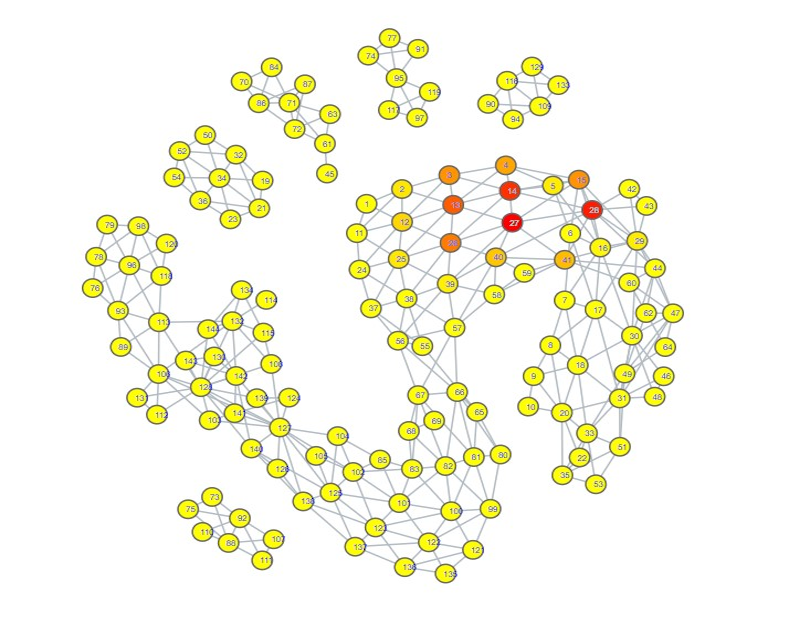

Supplement: S13 Fig — Yellow = No Suicidality Embeddings, Red = Entirely Comprised of Suicidality Embeddings. (TIFF) [file pdig.0000935.s013.tiff]

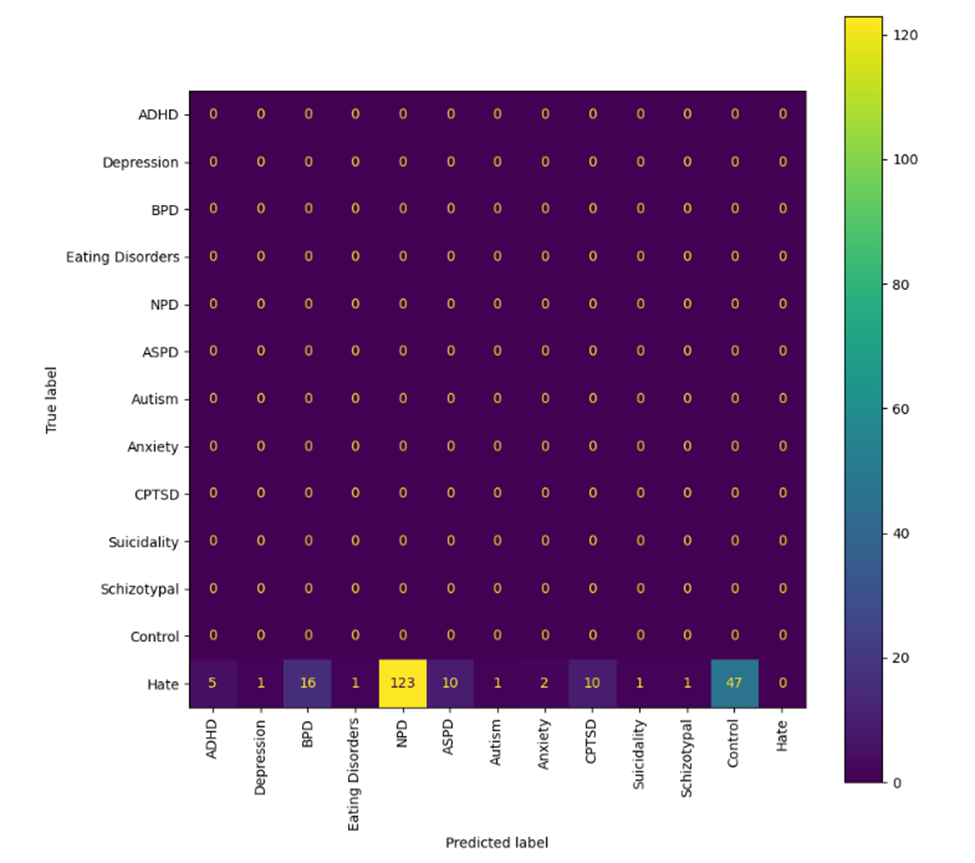

Supplement: S14 Fig — (TIFF) [file pdig.0000935.s014.tiff]

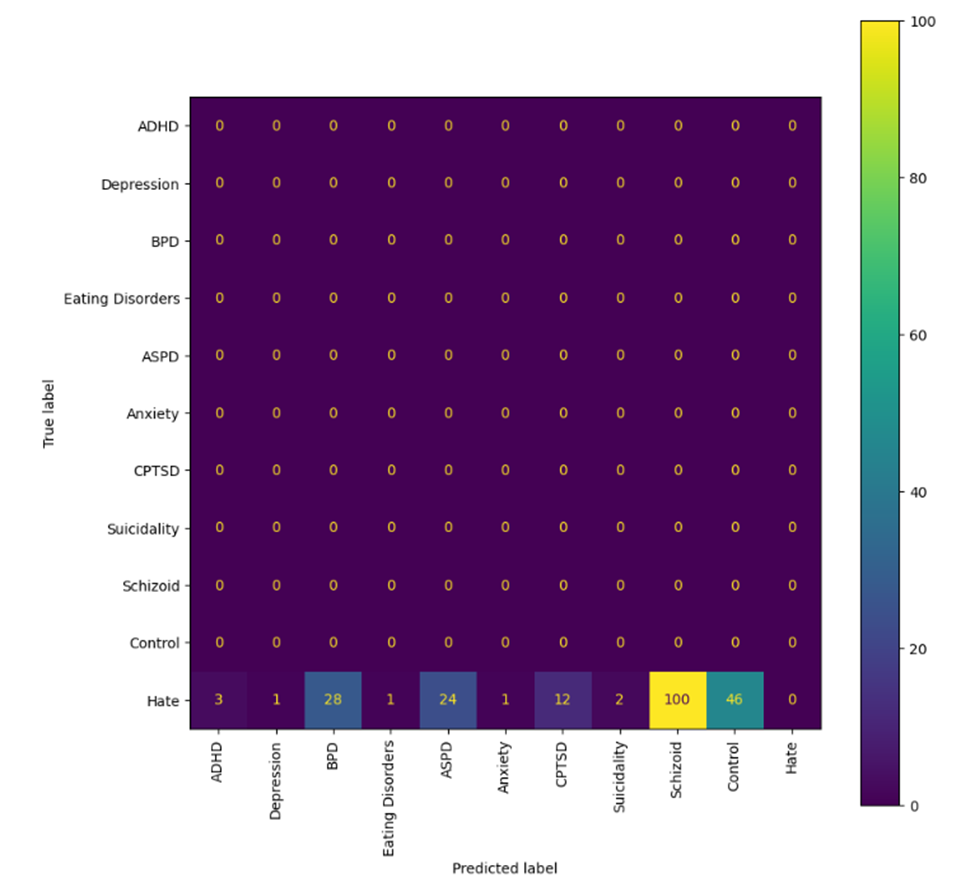

Supplement: S15 Fig — (TIFF) [file pdig.0000935.s015.tiff]

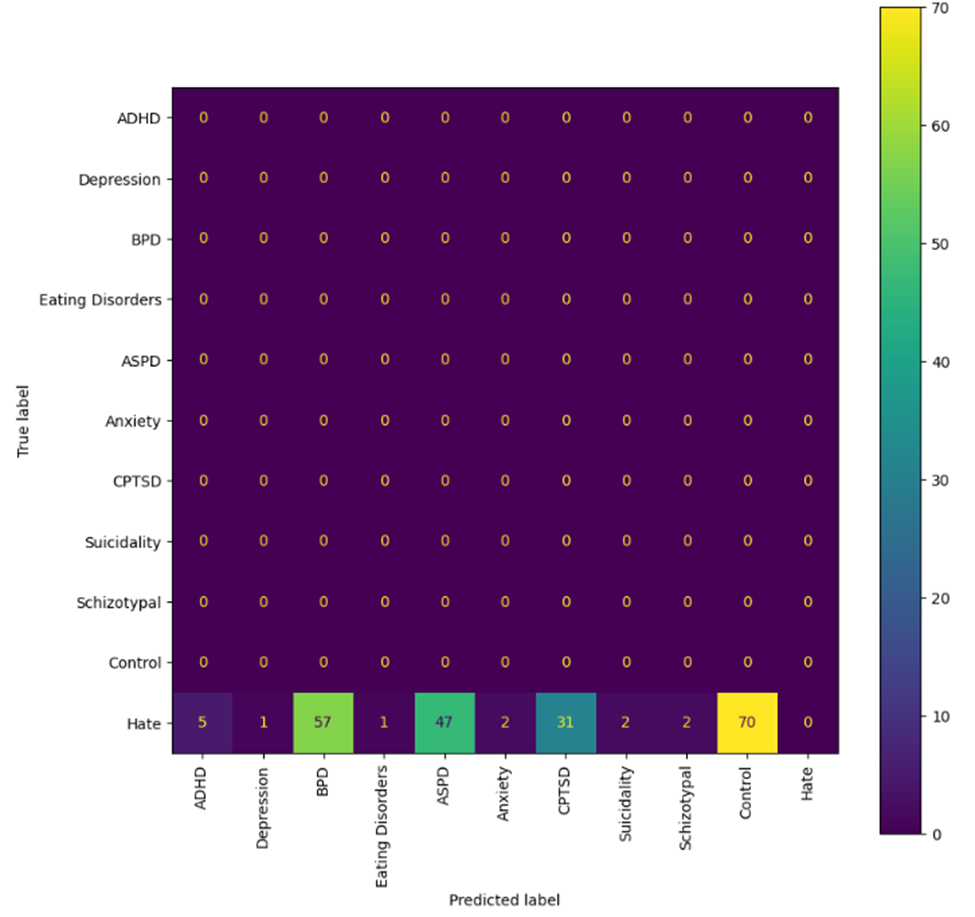

Supplement: S16 Fig — (TIFF) [file pdig.0000935.s016.tiff]

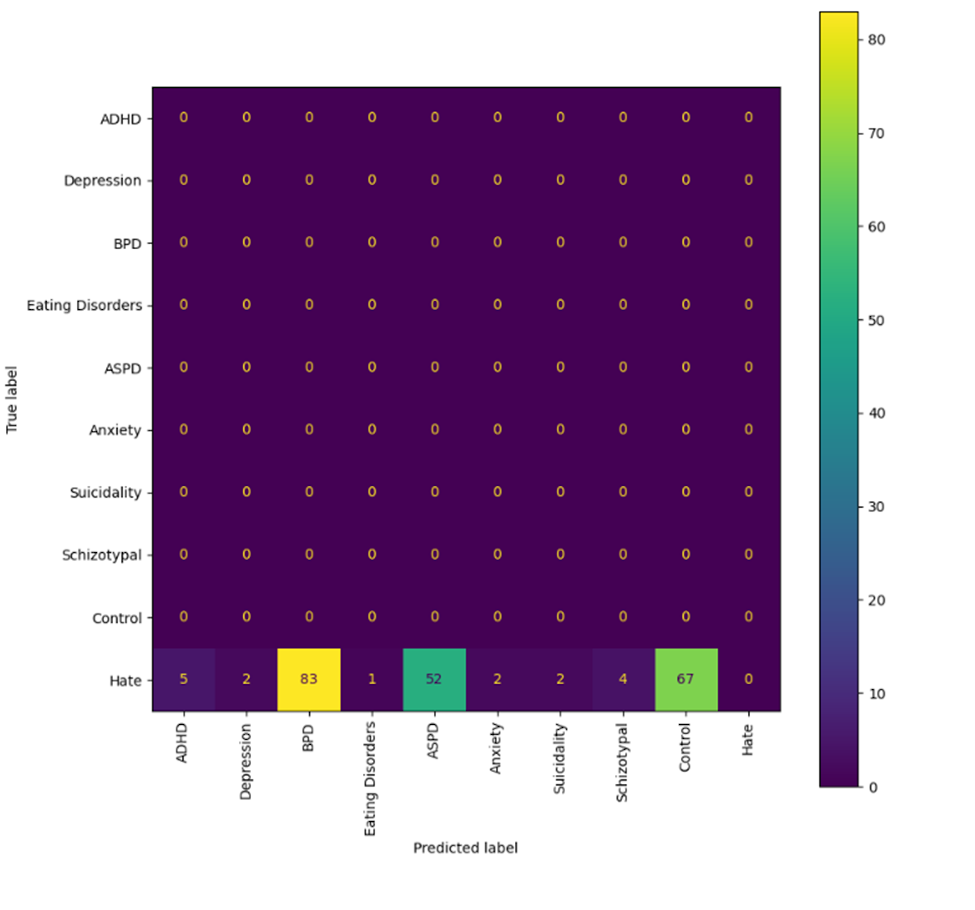

Supplement: S17 Fig — (TIFF) [file pdig.0000935.s017.tiff]

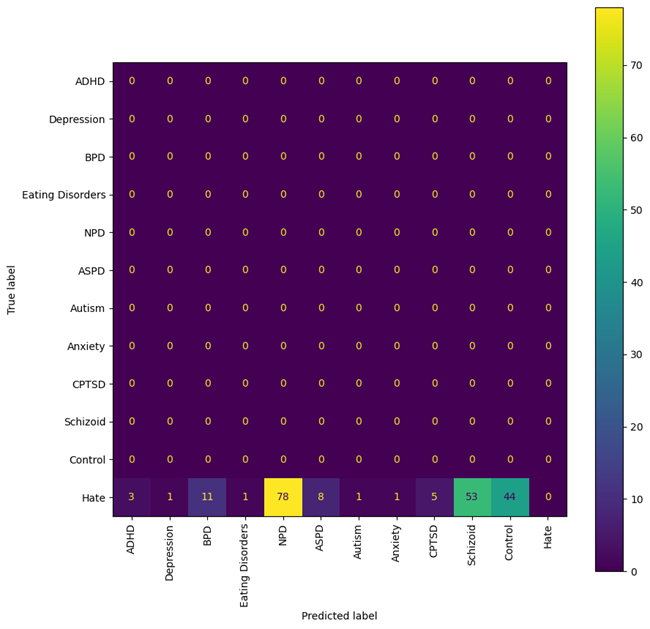

Supplement: S18 Fig — (TIF) [file pdig.0000935.s018.tif]

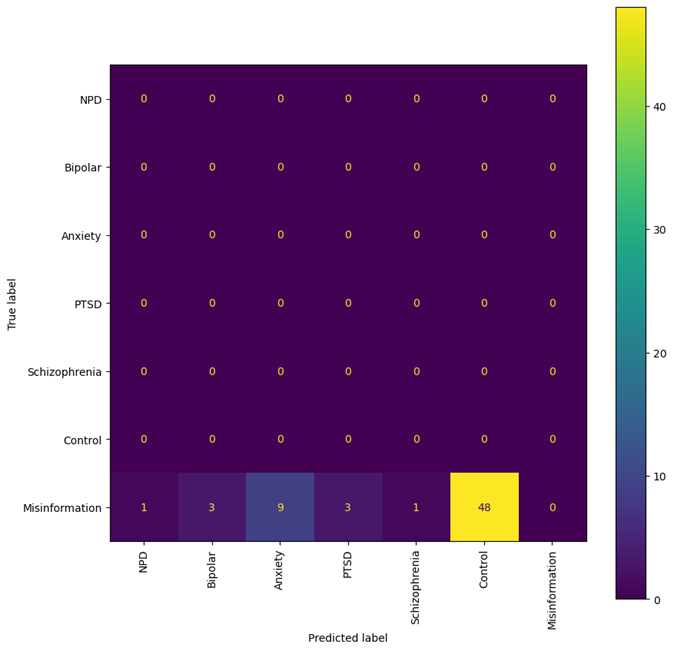

Supplement: S19 Fig — (TIF) [file pdig.0000935.s019.tif]
